# Sodium alginate-hydrogel coatings on extracorporeal membrane oxygenation for anticoagulation

**DOI:** 10.3389/fcvm.2022.966649

**Published:** 2022-11-01

**Authors:** Wenqing Gao, Han Wang, Yanwu Liu, Qin Tang, Peng Wu, Tingting Lin, Tong Li, Di Sun

**Affiliations:** ^1^Department of Cardiac Center, Tianjin Third Central Hospital, Tianjin, China; ^2^Tianjin Key Laboratory of Extracorporeal Life Support for Critical Diseases, Tianjin, China; ^3^Key Laboratory of Synthetic and Natural Functional Molecule of the Ministry of Education, College of Chemistry and Materials Science, Northwest University, Xi’an, China; ^4^Tianjin Key Laboratory of Retinal Functions and Diseases, Tianjin Branch of National Clinical Research Center for Ocular Disease, Eye Institute and School of Optometry, Tianjin Medical University Eye Hospital, Tianjin, China; ^5^Key Laboratory of Photochemical Conversion and Optoelectronic Materials, Technical Institute of Physics and Chemistry, Chinese Academy of Sciences, Beijing, China; ^6^CAS Key Laboratory for Biomedical Effects of Nanomaterials and Nanosafety, CAS Center for Excellence in Nanoscience, National Center for Nanoscience and Technology of China, Beijing, China

**Keywords:** hydrogel coating, ECMO tube, natural polysaccharide, sodium alginate, anticoagulation

## Abstract

Thromboembolism caused by the use of extracorporeal membrane oxygenation (ECMO) remains common among patients with existing heart diseases and contributes to significant morbidity and mortality during the COVID-19 pandemic. Various surface modification strategies have been proposed, showing that the methacrylated alginate (MA-SA) hydrogel layer is transparent, which aids the observation of the thromboembolism from the inner wall of the tubing. In the combined dynamic and static blood of ECMO tubing inner surface *in vitro* experiments, it was also demonstrated that the adhesion of blood clots to the surface of vessels was remarkably reduced, and the MA-SA-based hydrogel coating could significantly prolong the activated partial thrombin time and block the endogenous coagulation. The favorable properties of natural polysaccharides of hydrogel coatings make them the best surface material choices to be applied for blood-contacting medical devices and significantly improve anticoagulant performance.

## Introduction

Extracorporeal membrane oxygenation (ECMO) plays an important role during the COVID-19 pandemic. It is an extracorporeal lung assist technology used to partially or completely replace the patient’s cardiopulmonary function and extends the patient’s life while waiting for the primary disease to be treated. Membrane lungs, blood pumps, and blood pipelines are the core compositions of ECMO, they act as the artificial lung, heart, and blood vessels, respectively. Polyvinyl chloride (PVC) is among the raw materials of the blood pipeline, it ranked third among the widely produced plastic polymers worldwide (1).

For the cardiopulmonary bypass, the PVC circuit which can initiate the activation of platelets and the coagulation cascade after blood cell contact is a possible detrimental effect (2). The heparin-coated artificial anticoagulant is commonly used on the inner wall of the ECMO tubing to prevent blood clotting. The development of antithrombotic surfaces will be a major advancement in medical applications. The PVC surface activation was prepared on ammonia plasma-treated PVC (3).

Heparin was widely used as an anticoagulation coating because red blood cells (RBC) are negatively charged and RBC is repelling heparin (glycosaminoglycan with negative charge, Hep). Heparin is an animal-derived polysaccharide, which brings out animal sensitization. Natural polysaccharides exhibit anticoagulation mechanisms similar to heparin. It can be potentially developed into a natural anticoagulant and can be used as an alternative to heparin. Among polysaccharides, sodium alginate was selected as a non-toxic natural plant polysaccharide material, combined with calcium (coagulation factor IV) as an important component of anticoagulation function (4). Sodium alginate is a heparin-like polysaccharide, its sulfated polysaccharide site can bind to antithrombin III (AT-III), catalyze AT-III, antagonize coagulation factors IIa, Xa, IXa, XIa, and XIIa, thereby blocking the intrinsic coagulation pathway, inhibits the conversion of prothrombin to thrombin (IIa), inhibits thrombin activity, and hinders the conversion of fibrinogen to fibrin monomers.

Functional hydrogel coatings (5) play an essential role as structural components in the emerging field of medical devices by tailoring the molecular interactions between the hydrogel polymer network and drugs (i.e., covalent linkage, electrostatic interaction, and hydrophobic interaction) the drug release rate can be efficiently tuned (6–9). The materials used for the coating of sodium alginate are mainly PVC (10) and polyelectrolyte (PE) (11). At the same time, the latest hydrogel coating is also applied to the PVC tubing (12).

**FIGURE 1:**
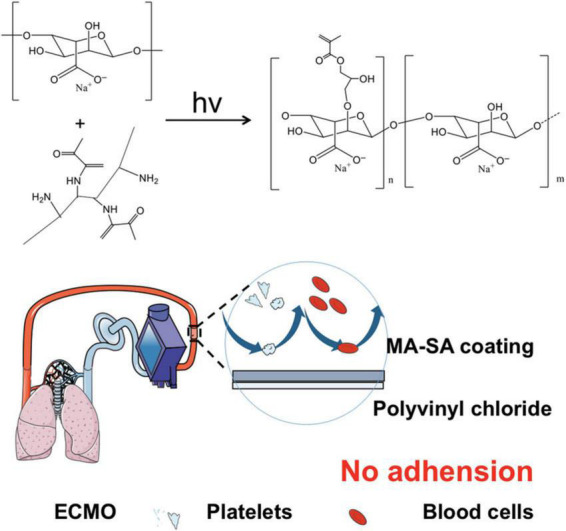
Methacrylated alginate (MA-SA)-based hydrogel coatings on extracorporeal membrane oxygenation (ECMO) surfaces. The medical device used in this study is polyvinyl chloride (PVC) tubing with MA-SA hydrogel-coated ECMO surfaces.

Thus, we synthesized the methacrylated alginate (MA-SA)-based hydrogel coating PVC tubing for anticoagulation with UV cross-link reaction, as illustrated in Figure 1.

## Materials

Alginate was purchased from Aladdin, Shanghai, China. Dimethyl sulfoxide was obtained from Beijing Chemical Reagent Company, Beijing, China. N-(3-aminopropyl) methacrylamide hydrochloride dimethyl sulfoxide and 1-hydroxybenzotriazole monohydrate were supplied by Sigma, USA. Phosphate buffer solution (PBS) was purchased from Biotopped, Beijing, China. Chloroform, isopropyl alcohol, and ethanol were obtained from Tianjin Chemical Reagent Wholesale Company, Tianjin, China. TRIzol Reagent was obtained from Ambion company, Austin, USA. DNase/RNase-Free Water was obtained from Soleibo Technology Co., Ltd., Beijing, China. IL-1β, IL-6, and TNF-α were obtained from General Biological Co., Ltd., Chuzhou, China.

### Preparation of the methacrylated alginate-based hydrogel coatings on extracorporeal membrane oxygenation surfaces

The synthesis of MA-SA-based hydrogel coating was carried out using the following procedure. After dissolving a total of 0.25 g of alginate in 75 ml of deionized water, a sufficient amount of N-(3-aminopropyl) methacrylamide hydrochloride was added to the alginate solution. Subsequently, a total of 20 ml of a 1:1 mixture of dimethyl sulfoxide and water containing 0.291 g of 1-ethyl-3-(3-dimethyl aminopropyl) carbodiimide hydrochloride and 0.205 g of 1-hydroxybenzotriazole monohydrate was added to the first solution. The resulting mixture was allowed to react at room temperature for 48 h. Then, the solutions were rinsed using a saturated soda solution and dialyzed against deionized water for 3 days (13).

The alginate and gelatin methacryloyl are chemically anchored on the ECMO tubing surfaces [Product Category: Disposable Extracorporeal Circulation Tube (Adult); Manufacturer: Dongguan Kewei Medical Equipment Co., Ltd.; Production batch number: 20190618]. ECMO tubing inner surfaces were cleaned using an ammonia plasma (3), washed with ethanol, and then completely dried. The aminosilane, a coupling agent for a silica-based material, functionalized the ECMO tubing and was further incubated in 1, 2, and 5 wt% MA-SA solution with 0.1% (w/v) Irgacure 2,959 under 365 nm ultraviolet light. The inner parts of the ECMO tubing were finally washed with deionized water and were completely dried before use (14).

### Properties of the methacrylated alginate-based hydrogel coatings on extracorporeal membrane oxygenation surfaces

The internal surface morphologies of the MA-SA-based hydrogel coatings were detected using a scanning electron microscope (SEM, HITACHI, Japan) after being completely lyophilized for 12 h. The static water contact angle measurement was performed at room temperature using a contact angle goniometer (DSA100, KRUSS, German). Using a microsyringe, a droplet (3 μL) of distilled water was dropped on the surface and the value of the water contact angle was recorded after 30 s.

### Compatibility of the methacrylated alginate-based hydrogel coatings on extracorporeal membrane oxygenation surfaces

#### Cell lines and cell culture

Human umbilical vein endothelial cells (HUVECs) were obtained from the American Type Culture Collection (ATCC). HUVECs were cultured in DMEM medium (Gibco, USA) supplemented with 10% of fetal bovine serum (FBS) (Gibco, USA) and 1% penicillin-streptomycin (HyClone, USA) in a humidified incubator (Thermo Fisher Scientific, USA) at 37°C and 5% CO_2_. The cultured medium was replaced in about 2∼3 days, and the cells grew to 80∼90% density for passage.

#### Cytotoxicity

Human umbilical vein endothelial cells (HUVECs) cells were cultured in DMEM medium (Gibco, USA) supplemented with 10% FBS (Gibco, USA) and 1% penicillin-streptomycin (HyClone, USA) in a humidified incubator (Thermo Fisher Scientific, USA) at 37°C and 5% CO_2_. The cytocompatibility of the MA-SA-based hydrogel coatings for ECMO was determined by a direct contact method between the MA-SA-based hydrogel and HUVECs. HUVECs were seeded on the inner tube surface of 1, 2, and 5 of the MA-SA-based hydrogel coatings ECMO at a density of 5 × 10^4^ cells/cm^2^ in 24-well plates and incubated for a day (10, 15).

#### Flow cytometry

Flow cytometry was used to test cell apoptosis. HUVECs cells were counted and then inoculated to 6-well plates. The same amount of medium was added to the NC group, meanwhile, the MA-SA-based hydrogel coating was added to the experimental group. After 24 h of treatment, cells were digested and collected with trypsin without EDTA (Solarbio, USA), and then washed twice with PBS (Gibco, USA). The collected samples were suspended in the binding buffer following the Annexin V-FITC/PI Apoptosis Detection Kit (KeyGEN BioTECH, China). After staining with Annexin V-FITC and PI in the dark at room temperature for 10 min, apoptotic cells were examined by flow cytometric analysis (BD Biosciences, USA) (Excitation wavelength Ex = 488 nm; Emission wavelength Em = 530 nm). The experimental results were analyzed with FlowJo version 10.6.2, and the average value was obtained from three independent experiments performed on each group.

### Human blood hemocompatibility assessment

Fresh whole blood was obtained from the Third Central Hospital of Tianjin, China using a standard vacuum blood collection tube. This procedure was approved by the Human Ethics Committee of the hospital (IRB-2020-025-01). The blood was then centrifuged at 3000 r/min for 10 min. The lower supernatant platelet-poor plasma (PPP) is used to assess clotting time. Wash the 0.5 mm × 0.5 cm MA-SA-based hydrogel coatings tubing in a 24-well plate thrice with distilled water. Subsequently, equilibrate the tube with PBS at 37°C for 30 min, and then add a total of 200 μl PPP and incubate at 37°C for 1 h. After incubation, the activated partial thrombin time (APTT), thrombin time (TT), prothrombin time (PT), and fibrinogen amount (FIB) of PPP were measured thrice using the automatic coagulation analyzer (Diagnostica Stago, STA-R Evolution, France).

### Investigation of the effects on anti-inflammatory gene expression of RAW cells

Total RNA was isolated with TRIzol reagent (Invitrogen) after 8 h induction with PVC and coating material. RNA was reverse transcribed by PrimeScript RT kit (TaKaRa). Quantitative RT-PCR was performed with SYBR Premix Ex Taq (TaKaRa) and QuantStudio 3 and 5 Real-time PCR (Thermo Fisher). Using the housekeeping gene β-actin as the baseline, the gene expression of PVC and coating material groups was quantitatively analyzed (16).

## Result and discussion

### The properties of the methacrylated alginate-hydrogel coatings

**FIGURE 2:**
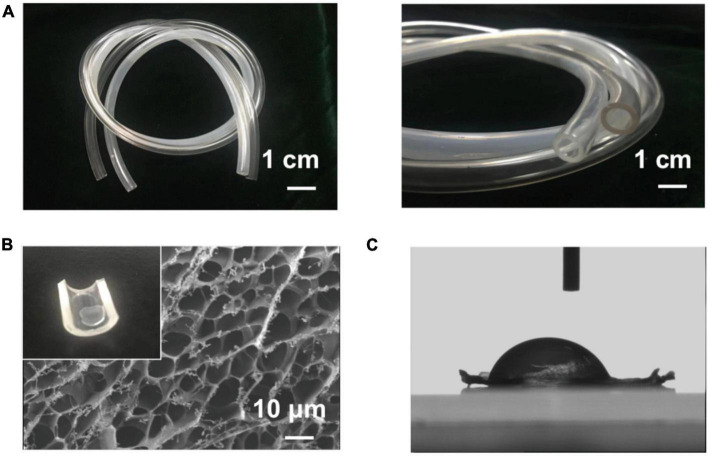
The image of methacrylated alginate (MA-SA)-hydrogel coatings on polyvinyl chloride (PVC) surfaces. **(A)** Macro images of the coating and the pristine tubing demonstrate that the method used here can produce a transparent and uniform hydrogel coating. **(B)** The scanning electron microscope (SEM) image of the methacrylated alginate (MA-SA)-hydrogel and the inset in the upper left corner of the hydrogel drop on the surface of the PVC tube. **(C)** The contact angle of MA-SA-hydrogel.

The surface of the MA-SA-hydrogel coatings was irradiated with UV light to polymerize the hydrogel layer. The MA-SA-hydrogel was shown in Supplementary Figure 1. Figures 2A,B demonstrated that the MA-SA hydrogel coating was uniform, illustrating macro images of pristine and the MA-SA-coated hydrogel tubing. The MA-SA-hydrogel coating is transparent and observing thrombosis through the tubing is easier than the N, N-dimethylacrylamide on the activated surfaces (17). The morphology of the MA-SA-hydrogel-coated ECMO surface is porous about 5 μm, however, the diameter of RBC observed in an optical microscope is less than 8 μm (18). The contact angle is 112° in Figure 2C, proving the MA-SA-hydrogel coating is hydrophobic, hence, a more favorable proof that the MA-SA-hydrogel coating is less prone to thrombosis.

### Anticoagulation properties of the methacrylated alginate-hydrogel-coated extracorporeal membrane oxygenation tubing to human blood

**FIGURE 3:**
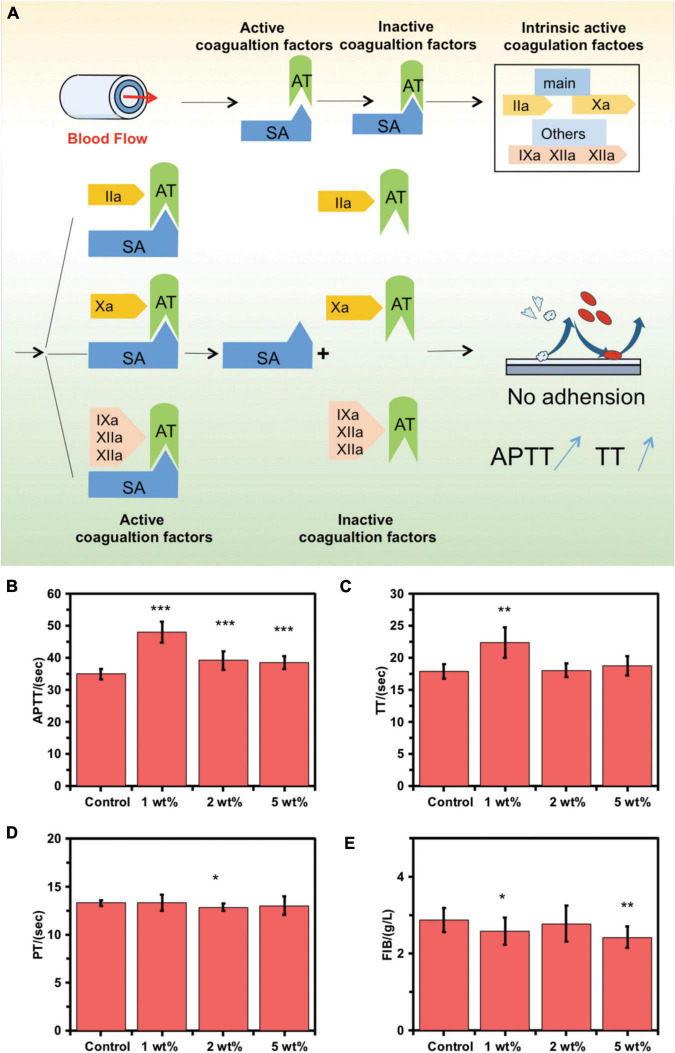
Coagulation function on the inner surface of control extracorporeal membrane oxygenation (ECMO) and MA-SA-Hydrogel coating tubings. **(A)** Schematic diagram of anticoagulation mechanism. **(B)** The activated partial thrombin time (APTT), **(C)** thrombin time (TT), **(D)** prothrombin time activity, and **(E)** fibrinogen amount (FIB) were measured using an automated blood coagulation analyzer and exposed to human blood (***p < 0.001; **p < 0.01; *p < 0.05; NS, not significant).

When blood passes through the inner surface of the MA-SA-hydrogel-coated tubing, the sodium alginate coating with a heparin-like structure binds with the antithrombin (AT) through a special pentose sequence, catalytically activates and amplifies AT activity. Activated AT binds to coagulation factors involved in the intrinsic coagulation pathway, and plays an anticoagulant effect by antagonizing the activated factor II (IIa) and factor X (Xa), including antagonizing IXa, XIa, and XIIa, thereby prolonging the APTT and TT. After tightly combining the AT-coagulation factor complex, it is separated from the surface of the sodium alginate coating and continues to play an anticoagulant effect in the blood while the fixed sodium alginate coating continues to bind and catalyze the activation of AT as shown in Figure 3A. Supplementary Figure 2 shows the good biocompatibility of the MA-SA-hydrogel coating.

The anticoagulant activity of the MA-SA-hydrogel-coated tubings was examined using the hemostasis indices, APTT, TT, PT, and FIB. Standard vacuum blood collection tubings were used to obtain patients’ wasted blood samples from healthy blood coagulation at Tianjin Third Central Hospital. Blood clots were formed when blood samples contact foreign surfaces following platelet activation. This can be catastrophic in clinical settings involving extracorporeal circulation, particularly during heart-lung bypass surgery where blood is circulated in PVC tubing (19). The hydrogel coating can significantly prolong the APTT and TT and block the intrinsic coagulation pathway, thereby significantly improving the anticoagulation performance of the inner surface of the ECMO tube in static blood *in vitro*. The APTT and TT in the 1 wt% MA-SA-hydrogel-coated tubings group were significantly increased (Figures 3B,C) as compared with those of the control ECMO tubing, indicating improved anticoagulation abilities with static blood inside the MA-SA-hydrogel-coated hydrogel tubing. No significant differences were observed in the PT (9.4–15.4 s) and FIB (2–4 g/l) (Figures 3D,E). The dexamethasone and oxidated sodium alginate mainly formed the composite coatings through ionic and covalent bond methods (20). SA/heparin composites were covalently immobilized onto the surface of the PVC pipeline (10). SA hydrogel sample with 1 wt% also has the best anticoagulant effect in our previous research (4). The same MA-SA-hydrogel-coated hydrogel coating treatment to the other component materials of the ECMO kit, polycarbonate (joint) and polymethylpentene (oxygenator membrane filament), and the anticoagulant properties of the two materials improved after coating in Supplementary Figure 3. Compared to protein-coated tubing (BioLine coating, MAQUET Inc.) and heparin coated tubing (Carmeda Bioactive Surface, Medtronic Inc.), sodium alginate-hydrogel coated PVC tubing also has anticoagulant properties and anticoagulant performance is slightly lower than commercial products. But, it is most important that sodium alginate comes from marine plants, which are more abundant and do not introduce animal-derived (heparin from animal intestinal mucosa) and human-derived (albumin from human blood) components, reducing the risk of allergy in patients in Supplementary Figure 4. Uncoated PVC tubing can activate the inflammatory response, thereby increasing the release of pro-inflammatory cytokines IL-6 and TNF-α from macrophages, and coated PVC tubing can reduce the release of IL-6 and TNF-α from macrophages, thereby reducing inflammation reaction in Supplementary Figure 5.

### Anticoagulation properties of the methacrylated alginate-hydrogel-coated extracorporeal membrane oxygenation tubing to simulated blood

**FIGURE 4:**
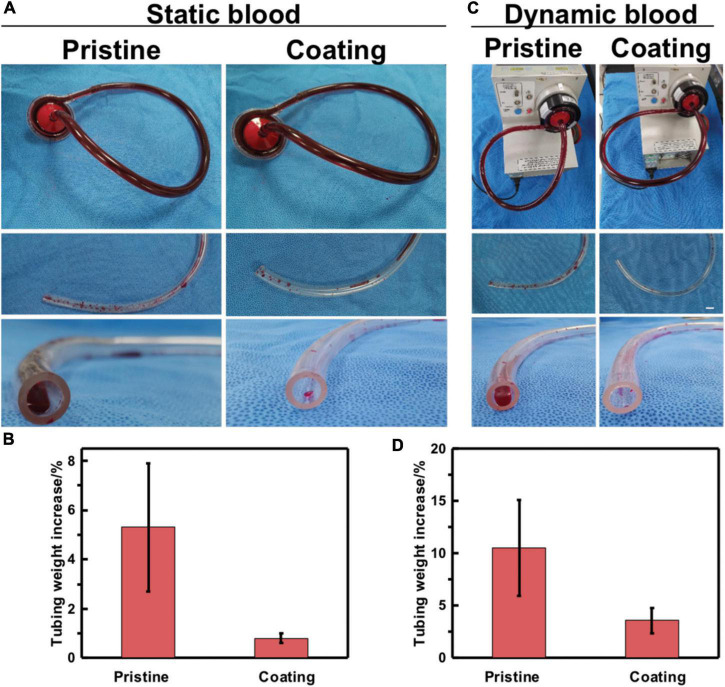
*In vitro* blood loop tests of static blood and dynamic blood. **(A)** Image of apparatus used for continuous flow testing of pristine and coated tubing. Testing was done in parallel to accurately control the end time point of flow. **(B)** Quantification of blood clotting adhesion to the tubing walls in static blood. **(C)** Image of pristine and coated tubing after flow testing and gentle rinsing with saline. **(D)** Quantification of blood clotting adhesion to the tubing walls in dynamic blood. Error bars represent SD for three repeated experiments.

The control blood sample in closed circular tubing loops was circulated until it is fully coagulated, as shown in Figure 4A. Simulated blood that was circulated through pristine tubing formed larger amounts of wall-adhering clots than blood in contact with coated tubing. The clotted blood samples were subsequently poured into a petri dish, blood clots were removed and weighed. Then, the tubing was gently rinsed twice and then weighed. The average pristine tubing weight gain is 5.3%, whereas that of the coated tubing is 0.8% in static blood, as shown in Figure 4B.

Whole blood was circulated through the circuit using a cardiopulmonary bypass circuit machine roller pump at a flow rate of 2.5–3.5 L/min, as shown in Figure 4C. In dynamic blood, the average tubing weight gain for the pristine tubing was 10.5% as compared to the coated tubing in Figure 4D which has a 3.6% weight gain. This indicates that blood clots adhered more readily to the pristine than to the MA-SA-coated tubing. These two pieces of evidence indicate that the MA-SA-hydrogel coatings on the PVC tubing show potential benefits in medical applications to address thrombosis-related complications.

## Conclusion

These experiments show evidence that surface modification of the PVC with SA hydrogel coating leads to platelet activation, thrombosis, and blood incompatibility. The MA-SA-hydrogel coatings decreased the adhesion and activation of platelets thus improving their anticoagulation performance. It is believed that hydrophobic materials and natural polysaccharide surfaces of the coated tubing prolong the APTT and TT thereby blocking the intrinsic coagulation pathway. The MA-SA-hydrogel-coating technology is a design strategy that may mitigate the thromboembolism caused by the use of blood-contacting medical devices.

## Data availability statement

The original contributions presented in this study are included in the article/Supplementary material, further inquiries can be directed to the corresponding authors.

## Ethics statement

The studies involving human participants were reviewed and approved by the Third Central Hospital of Tianjin, China IRB-2020-025-01. The patients/participants provided their written informed consent to participate in this study.

## Author contributions

WG and DS: conceptualization, investigation, and writing – original draft preparation. WG, DS, HW, YL, QT, PW, and TiL: methodology. YL, TiL, and DS: validation. QT and DS: data curation. WG, DS, and ToL: writing – review and editing and funding acquisition. All authors have read and agreed to the published version of the manuscript.
