# Supplementary material for: Sodium alginate-hydrogel coatings on extracorporeal membrane oxygenation for anticoagulation
Source: Front Cardiovasc Med. 2022 Nov 1;9:966649. doi: 10.3389/fcvm.2022.966649 (PMC9663475; doi:10.3389/fcvm.2022.966649)
Supplement: Supplementary file 1 [file Data_Sheet_1.docx]

**Support Information**

**
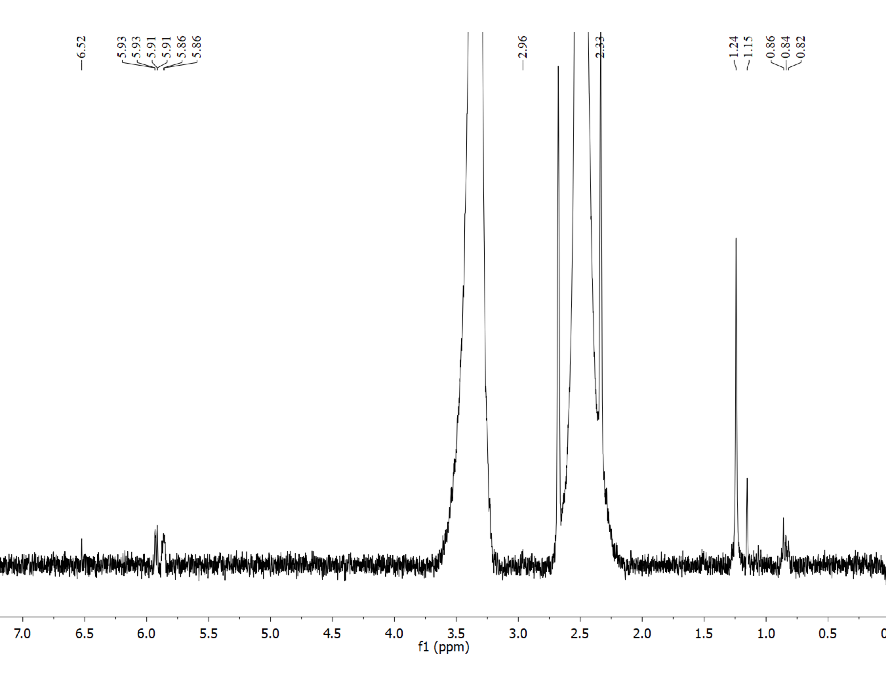
**

Figure. S1. ^1^H NMR spectra (400 MHz) of the MA-SA hydrogel in DMSO-d6.

**
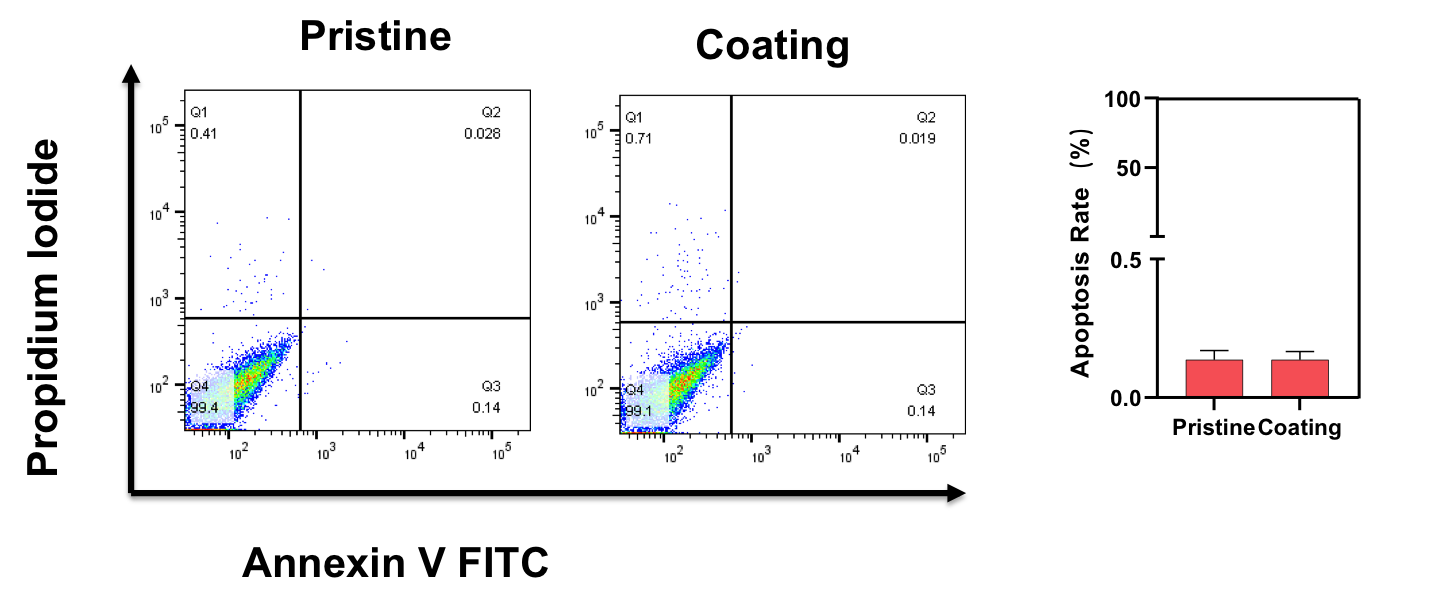
**

Figure. S2. Flow cytometry of was carried out to test cell apoptosis, which was quantified as the apoptosis rate. No significant difference was found between each group (p > 0.05).


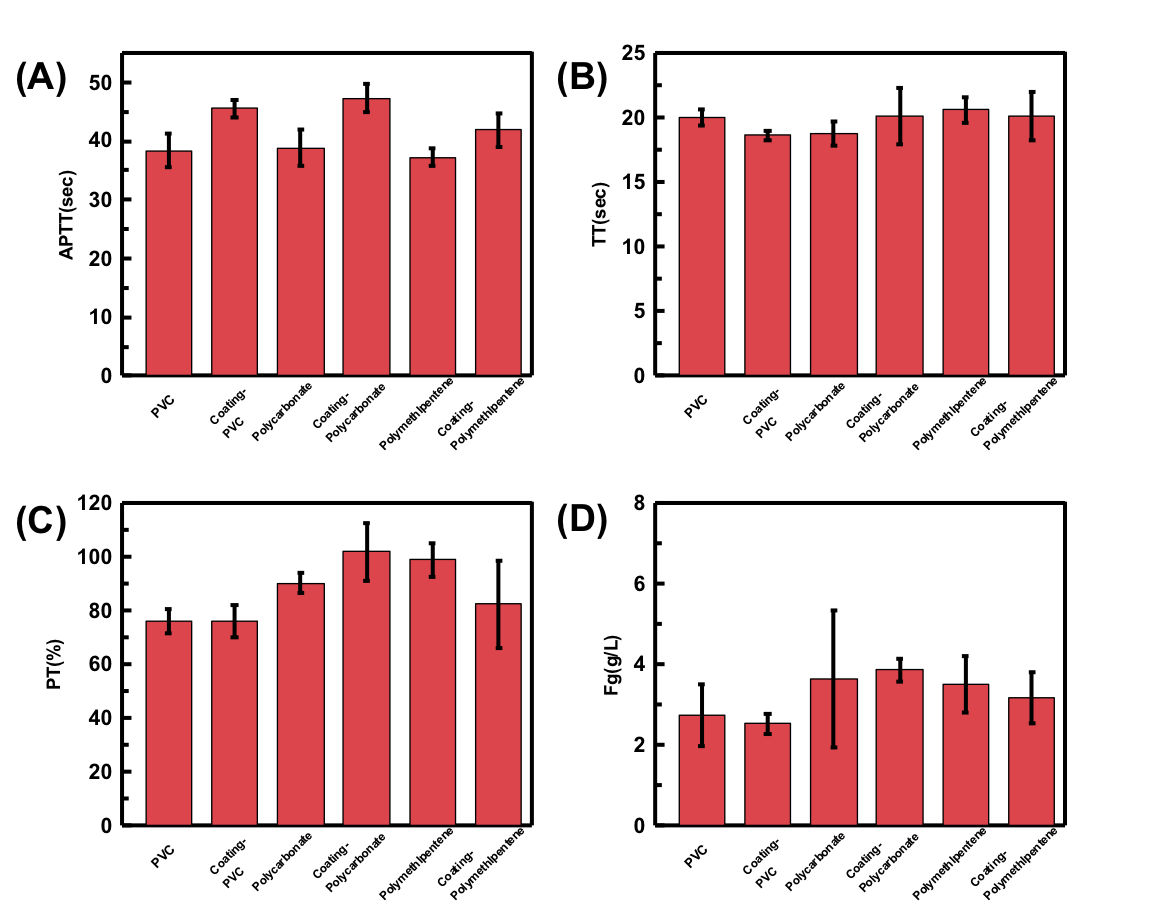


Figure.S3 Coagulation function on the inner surface of PVC and MA-SA-Hydrogel coating PVC, polycarbonate, MA-SA-Hydrogel coating polycarbonate, polymethylpentene, and MA-SA-Hydrogel coating polymethylpentene. (A) The activated partial thrombin time (APTT), (B) thrombin time (TT), (C) prothrombin time activity, and (D) fibrinogen amount (FIB) were measured using an automated blood coagulation analyzer and exposed to human blood (****P* < 0.001; ***P* < 0.01; **P* < 0.05; NS, not significant). Note: PVC(tubing) and Polycarbonate(fittings) are from the manufacturer: Dongguan Kewei Medical Equipment Co., Ltd., product category: Disposable Extracorporeal Circulation Tube (Adult), production batch number: 20190618. Polymethylpentene is from manufacturer: Japan's Mitsui Chemicals; product Category: PTX-MX002.


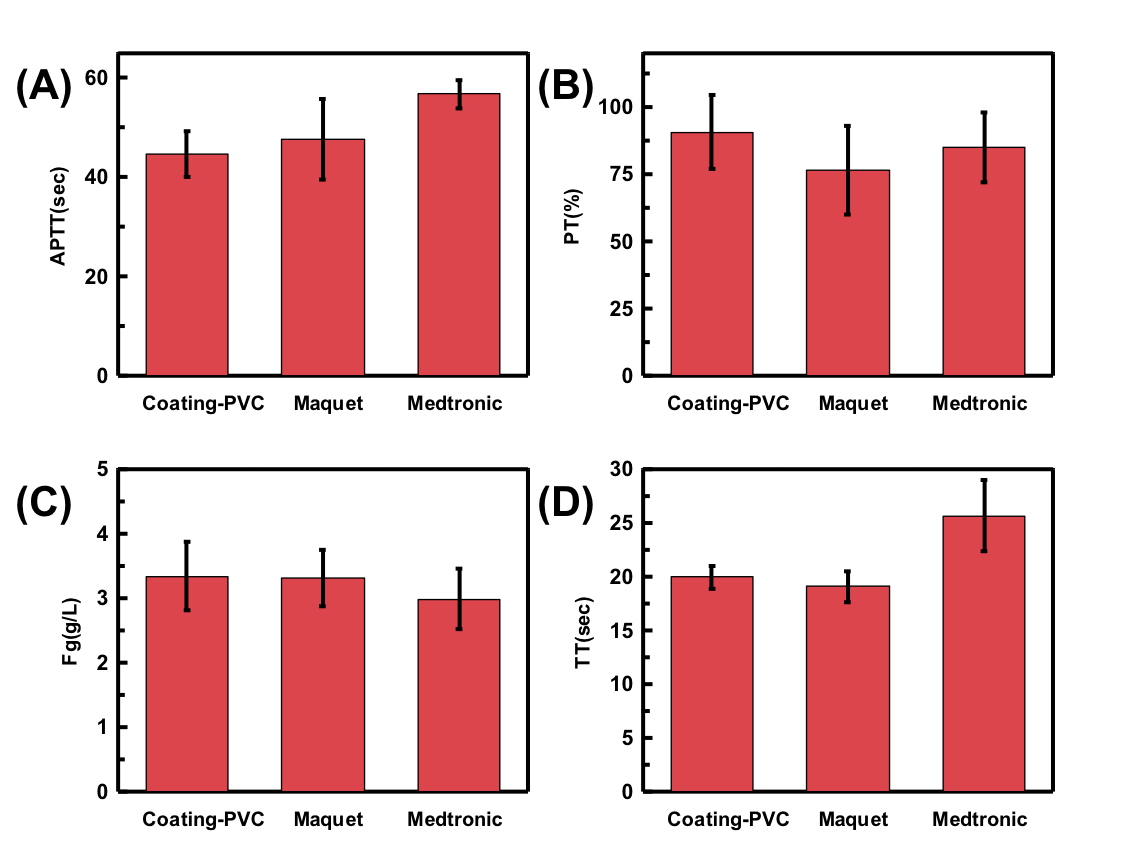


Figure.S4 Coagulation function on the inner surface of MA-SA-Hydrogel coating ECMO, Maquet brand ECMO, and Medtronic brand ECMO. (A) The activated partial thrombin time (APTT), (B) thrombin time (TT), (C) prothrombin time activity, and (D) fibrinogen amount (FIB) were measured using an automated blood coagulation analyzer and exposed to human blood (****P* < 0.001; ***P* < 0.01; **P* < 0.05; NS, not significant). Note: protein-coated tubing (BioLine coating and heparin-coated tubing (Carmeda Bioactive Surface, Medtronic Inc), MAQUET Inc)


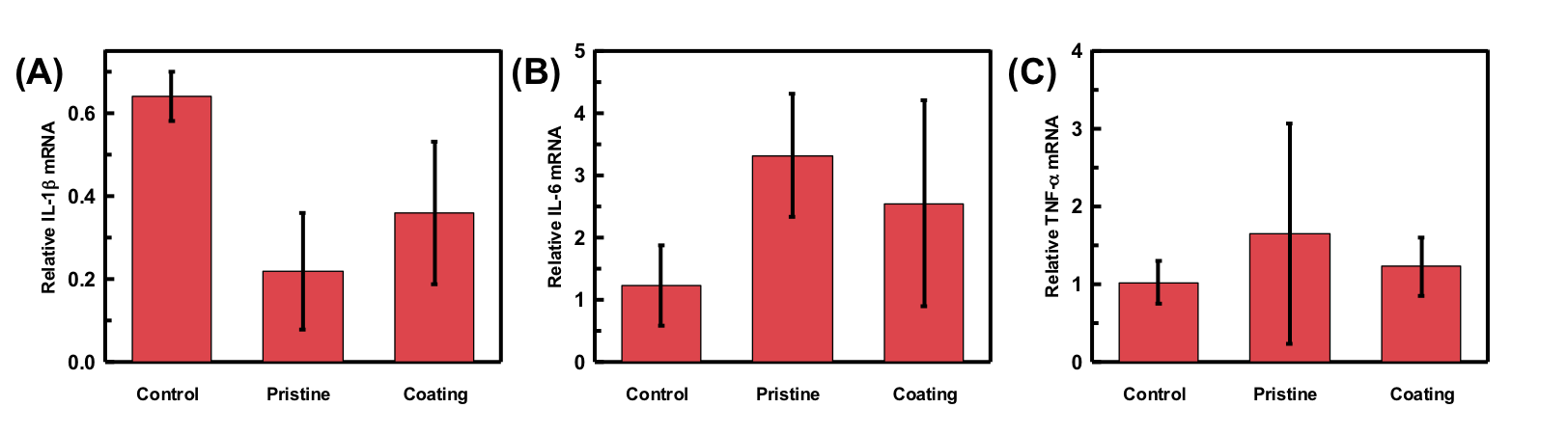


Figure. S5. Gene expressions of IL-1β, IL-6, and TNF-α in RAW cells cultured with PVC tubing (pristine) and MA-SA-hydrogel coatings on PVC surfaces(Coating).
